# Cranberry Oil: A Potent Natural Intimate Care Ingredient Displaying Antioxidant and Anti-Inflammatory Effects and Promoting Beneficial Vaginal *Lactobacillus*

**DOI:** 10.3390/ijms26052176

**Published:** 2025-02-28

**Authors:** Cloé Boira, Julia Jolibois, Anaïs Durduret, Jean Tiguemounine, Caroline Szewezyk, Morgane De Tollenaere, Amandine Scandolera, Romain Reynaud

**Affiliations:** 1 Givaudan Active Beauty, R&D, 51110 Pomacle, France; cloe.boira@givaudan.com (C.B.); anais.durduret@givaudan.com (A.D.); morgane.de_tollenaere@givaudan.com (M.D.T.); amandine.scandolera@givaudan.com (A.S.); 2 Polyclinique Courlancy, Surgery, 51100 Reims, France; jean.tigue@me.com; 3 Givaudan France Naturals, R&D, 84911 Avignon, France; caroline.szewezyk@givaudan.com; 4 Givaudan Active Beauty, R&D, 31400 Toulouse, France; romain.reynaud@givaudan.com

**Keywords:** skin, epithelium, keratinocytes, *Vaccinium macrocarpon*, cranberry, fatty acids, *Candida albicans*, *Gardnerella vaginalis*, *Lactobacillus*, intimate care, vaginal flora

## Abstract

Cranberry oil is known for nutritional benefits, and this work is aimed at studying its soothing properties and potential as an intimate care ingredient. The antioxidant, anti-inflammatory, and anti-irritation properties of cranberry oil were evaluated on epithelial cells and tissues, including the vaginal epithelium. The impact of the oil on vaginal microbiota was assessed in vitro. Cranberry oil reduced oxidative stress in keratinocytes (ROS −43%) and lowered inflammation by lessening the release of cytokines IL-8 (−33%) and TNF-α (−32%). Irritation induced by sodium dodecyl sulfate (SDS) in skin explants was lowered by 24%. Cranberry oil and fruit extract acted synergistically on inflammation, decreasing TNF-α release by 75% (vs. −34% and −16%, respectively). Cranberry oil reduced inflammation on EpiVaginal™ tissue, decreasing IL-6 by 36%. The minimum inhibitory concentration (MIC) of cranberry oil on the pathogenic vaginal microorganisms *C. albicans* and *G. vaginalis* was 0.5% and 0.1%, respectively. The oil promoted the growth of commensal *L. jensenii* (×79 at 0.1%) and favored a high proportion of lactic acid bacteria when co-cultured with *C. albicans*. Cranberry oil has antioxidant, anti-inflammatory, and soothing properties on skin. Anti-inflammatory activity was confirmed on vaginal epithelium, and initial in vitro evidence indicates that the oil can balance vaginal flora to prevent dysbiosis.

## 1. Introduction

Cranberry (*Vaccinium macrocarpon* Aiton) is considered a superfood thanks to its high bioactive compound content, particularly antioxidant and anti-inflammatory flavonoids. Cranberry consumption can prevent several pathologies: urinary tract infections, cystitis, oxidative stress, cardiovascular diseases, obesity, type 2 diabetes, *Helicobacter pylori* infection, cancer, and periodontitis [1,2,3]. The red color of berries is due to the presence of anthocyanins and their aglycones and anthocyanidins, both of which have antioxidant and anti-inflammatory properties. Berries are also rich in proanthocyanidins, flavonols, mainly quercetin and myricetin glycosides, and triterpenoids, with the major one being ursolic acid [4]. Cranberry fruit extract has demonstrated antioxidant and antibacterial properties and it exerts cytotoxic activity on cancer cell lines [5].

The antibacterial activity of cranberry fruit extract is related to its phenolic compounds, with both polar and apolar fractions being able to decrease microbial growth [6,7]. Nevertheless, other compounds such as sugar and organic acids seem to contribute to the antibacterial activity of cranberry [8]. In the context of increasing antimicrobial resistance, natural compounds represent an alternative to antibiotics in the prevention or management of simple infections [9]. Consumption of cranberries is recommended for prophylaxis of urinary tract infections [10,11]. This is supported by the antiadhesive properties of polyphenols, carbohydrates, terpenes, and organic acids from cranberries, preventing adherence of pathogens such as *Escherichia coli* to uroepithelial cell receptors. A more recent hypothesis suggests that cranberry consumption can modify gut microbiota composition and indirectly influence the urinary microbiota balance. Maintaining a healthy microbiome balance appears to be a therapeutic strategy, and the combination of cranberry extract and *Lactobacillus* probiotics is currently proposed. Cranberry fruit extracts and urinary metabolites derived from cranberry consumption not only display antibacterial activity but also have antiadhesive activity against the fungus *Candida albicans*, which is involved in genitourinary infections [12,13]. Bacterial vaginosis and vulvovaginal candidiasis are common vaginal dysbiosis conditions characterized by malodorous discharge and irritation [14]. The recurrence of vulvovaginal infections after antibiotic treatment highlights the importance of microbiota in the management of this epidemiological problem, which can lead to complications like infertility, pre-term birth, and other infectious diseases [14]. A healthy vaginal microbiota is generally described as predominated by *Lactobacillus* species such as *L. crispatus* or *L. jensenii*, while dysbiosis is characterized by bacteria including *Gardnerella vaginalis* [14]. Probiotic lactobacilli are already used in the prevention and treatment of vaginal infections [15]. Recent findings have also demonstrated the potential of oleic acid and similar long-chain fatty acids in the treatment of bacterial vaginosis [16]. Oleic acid enhances the growth of *L. crispatus* and decreases dysbiosis-associated *L. iners* thanks to enzymatic equipment that biochemically sequesters the fatty acid and makes it available only for *Lactobacilli* expressing the associated genes. Fatty acids are growth factors for *Lactobacilli*, and polyunsaturated fatty acids have also been demonstrated to promote bacterial adhesion to the intestinal mucosa [17,18]. Moreover, some polyunsaturated fatty acids exhibit antibacterial properties, such as linoleic acid, disturbing cell wall biosynthesis in *E. coli* or *Staphylococcus aureus* [19].

The agro-industrial transformation of cranberry offers byproducts, including those that are a source of fatty acids. Cranberry oil is rich in oleic acid and displays a unique profile of omega-3, omega-6, and omega 9 polyunsaturated fatty acids in the form of triacylglycerols: 21 to 35% of α-linolenic acid (C18:3 n-3), 36–39% of linoleic acid (C18:2 n-6) and 20–24% of oleic acid (C18:1 n-9) [20,21,22,23,24,25,26,27]. On top of the role of polyunsaturated fatty acids in managing vaginal dysbiosis, omega-3 fatty acids and their metabolites exhibit anti-inflammatory properties that could be beneficial in alleviating the consequences of infections, such as itching [28]. Other bioactive compounds from cranberry oil are scarcely studied, although its composition differs significantly from the pericarp composition. Tocochromanols were identified in seeds, especially γ- and δ-tocotrienols, together with tocopherols [21,25]. Phytosterols were also detected in significant quantities in oil and seed extract, mainly in the form of campesterol, β-sitosterol, β-amyrin, and squalene [4,25]. Seed extract also contains triterpenes in the form of oleanolic acid and ursolic acid [4]. Seeds are differentiated from other fruit parts (peel, pulp) by their high content of campesterol, squalene, and, to a lesser extent, β-amyrin and β-sitosterol. Several recent studies indicate campesterol and β-sitosterol to be potent antimicrobial compounds, and campesterol was shown to present potentiating activity of antibiotics against multiresistant strains of *S. aureus* [29,30,31]. Cranberry oil also contains phenolic compounds, quantified in gallic acid equivalents as around 320 mg/100 g [32]. The biological properties of cranberry oil are understudied. Shivananda Nayak and colleagues demonstrated that cranberry oil stimulates skin wound healing in a rat wound model and suggested that this activity could be attributed to the antioxidant activity of polyphenols or omega-6 and omega-9 fatty acids [32]. The authors also observed that cranberry oil exerts antibacterial activity on *Escherichia coli*, *Staphylococcus aureus*, and *Klebsiella pneumoniae*.

In this work, we aimed to characterize the soothing properties of *V. macrocarpon* oil to alleviate skin itching and inflammation. Indeed, epithelial irritation is triggered by physical factors (temperature, pollution, shaving, etc.), chemicals (detergents, latex, dyes, etc.), or pathologies (vulvovaginal infections, eczema, psoriasis). The antioxidant, anti-inflammatory, and anti-irritation activities of cranberry oil were evaluated in human epithelial cells and tissues, including a vaginal skin model. The synergistic activity of cranberry oil and fruit extract on inflammation was tested. Anti-irritation activity was also characterized on skin explants. To further pursue the interest of cranberry oil for intimate care, its effect on the growth of vaginal pathogens and commensal bacteria was evaluated on individual species and also in co-culture.

## 2. Results

### 2.1. Cranberry Oil Decreases TBP-Induced ROS on Keratinocytes

**Figure 1:**
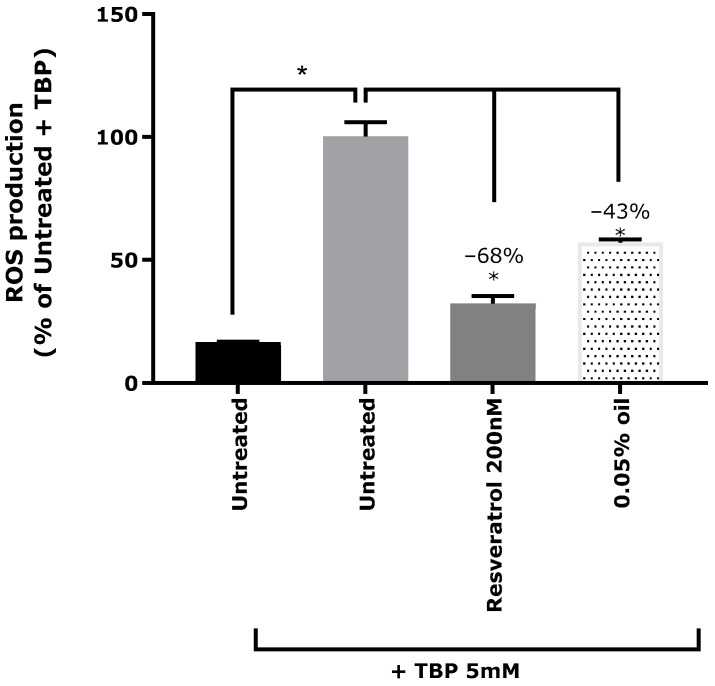
ROS (reactive oxygen species) production in normal human epidermal keratinocytes. Oxidative stress was induced with TBP at 5 mM (tert-Butyl hydroperoxide), and keratinocytes were pre-treated with the antioxidant reference molecule, resveratrol at 200 µM (positive control), or with cranberry oil at 0.05%. ROS production is expressed in percentage of TBP-treated condition. Mann–Whitney U-test, * *p* < 0.05.

Oxidative stress was induced in Normal Human Epidermal Keratinocytes (NHEKs) with tert-Butyl hydroperoxide (TBP) solution at 5 mM, as demonstrated by the induction of ROS by 83% compared to the untreated keratinocytes (Figure 1). Pre-incubation with resveratrol at 200 µM, a known natural antioxidant compound, used here as a positive control, decreased reactive oxygen species (ROS) by 68% compared to cells treated with TBP but not pre-incubated. Cranberry oil at 0.05% displayed antioxidant activity on keratinocytes, significantly decreasing ROS production by 43% (*p* < 0.05).

### 2.2. Cranberry Oil Decreased PMA-Induced IL-8 and TNF-α on Keratinocytes

**Figure 2:**
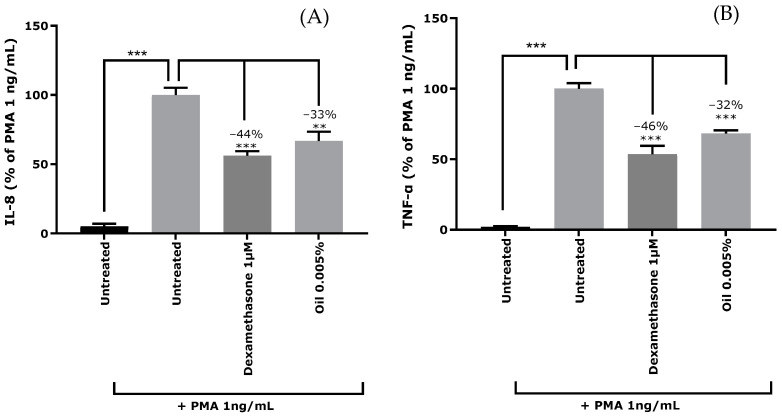
Release of (**A**) cytokines IL-8 (Interleukin 8) and (**B**) TNF-α (Tumor Necrosis Factor alpha) (**B**) in normal human epidermal keratinocytes stressed with PMA (Phorbol 12-Myristate 13-Acetate) and pre-treated with the anti-inflammatory reference compound dexamethasone at 1 µM (positive control) or cranberry oil at 0.005%. Quantification of cytokines is expressed as a percentage of the response of PMA treated condition. Mann–Whitney U-test, ** *p* < 0.01, *** *p* < 0.001.

Inflammation was triggered in NHEKs by Phorbol 12-Myristate 13-Acetate (PMA) at 1 ng/mL, as demonstrated by the release of IL-8 (Figure 2A) and TNF-α (Figure 2B) cytokines in culture medium. Pre-treatment with dexamethasone, a synthetic anti-inflammatory corticoid, decreased IL-8 release by 44% and TNF-α by 46%. Cranberry oil at 0.005% exhibited an anti-inflammatory activity on keratinocytes, significantly decreasing IL-8 levels by 33% and TNF-α by 32%.

### 2.3. Cranberry Oil Decreased Flagellin-Induced IL-6 on Reconstructed Human Vaginal Epithelium

**Figure 3:**
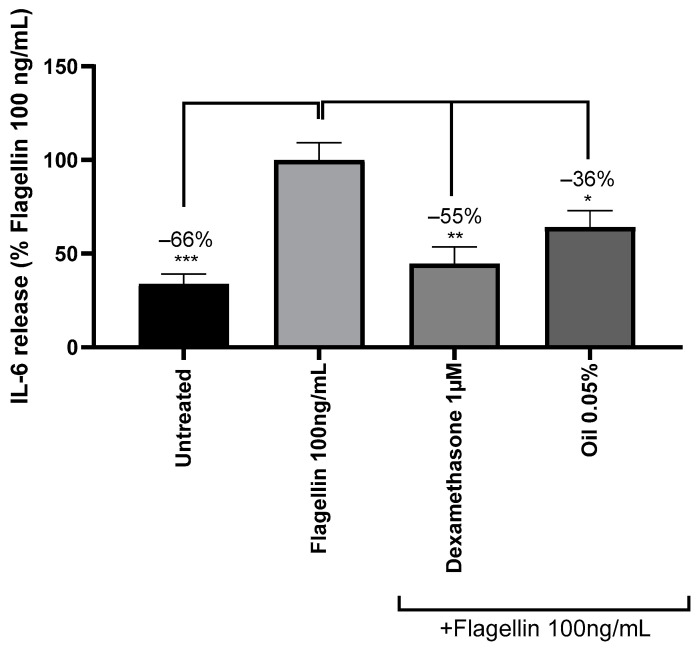
Release of cytokine IL-6 (Interleukin 6) EpiVaginal™ tissues stressed with flagellin 100 ng/mL and pre-treated with the anti-inflammatory reference compound dexamethasone at 1 µM (positive control) or cranberry oil at 0.05%. Quantification of IL-6 is expressed as a percentage of the response of flagellin-treated condition. Mann–Whitney U-test, * *p* < 0.5, ** *p* < 0.01, *** *p* < 0.001.

Flagellin is a bacterial protein involved in cell mobility that interacts with receptors of immune cells [33]. In this study, flagellin was used to mimic inflammation associated with bacterial vaginosis, on a reconstructed human vaginal epithelium, Epivaginal™, based on normal human-derived vaginal–ectocervical epithelial and dendritic cells. Treatment with 100 ng/mL of flagellin significantly increased the release of the cytokine IL-6 by the EpiVaginal™ tissues, suggesting an inflammatory response. Pre-treatment with the positive reference, dexamethasone, significantly reduced IL-6 by 55%. Pre-incubation with cranberry oil at 0.05% significantly reduced the IL-6 release by 36% (Figure 3), indicating that it could alleviate inflammation in the context of bacterial vaginosis.

### 2.4. Cranberry Oil Decreased Chemical Irritation on Skin Explants

Chemical detergents and microbiota dysbiosis can trigger inflammation in the skin and vulvovaginal tissue, and lead to several symptoms including irritation [14,34]. This condition was modeled on skin explants by applying sodium dodecyl sulfate (SDS) 0.2%. An irritation scoring based on histochemical observation was developed to quantify *stratum corneum* alteration (barrier function), edema, and dermal–epidermal junction cohesion.

**Figure 4:**
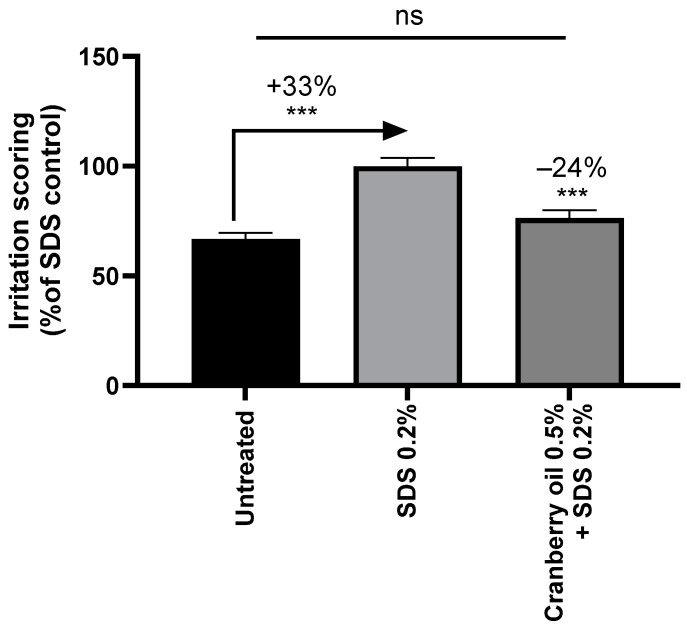
Irritation scoring on skin explants stressed with SDS at 0.2% (sodium dodecyl sulfate). Mann–Whitney U-test, *** *p* < 0.001, ns not significant.

**Figure 5:**
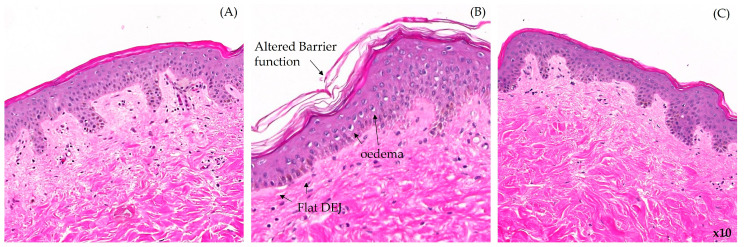
Morphological aspect of the skin explants: (**A**) untreated; (**B**) treated with SDS at 0.2% (sodium dodecyl sulfate) to induce irritation characterized by an altered barrier function, edema, and flat dermal–epidermal junction (DEJ), indicated by arrows; and (**C**) pre-treated with cranberry oil 0.5% before irritation induction with SDS at 0.2%. Skin explant slices from a 45-year-old woman were treated with hematoxylin and eosin staining for microscopic observation (magnification x10). All images shown are from the same donor.

Application of cranberry oil at 0.5% decreased, by 24%, the consequences of chemical irritation (Figure 4). Figure 5 provides illustrative pictures of the histochemical analysis of untreated explants (A), SDS-treated explants (B), and SDS + cranberry oil-treated explants (C). Cranberry oil significantly reduced irritation signs and restored a normal morphology of the skin.

### 2.5. Cranberry Oil Decreases Growth of Pathogenic Microorganisms and Promotes Commensal Lactic Bacteria of the Vulvovaginal Flora In Vitro

The impact of cranberry oil was evaluated on the growth of common microorganisms of the vulvovaginal flora, considered pathogenic: *Candida albicans*, *Gardnerella vaginalis*; or commensal: *Lactobacillus jensenii*.

**Figure 6:**
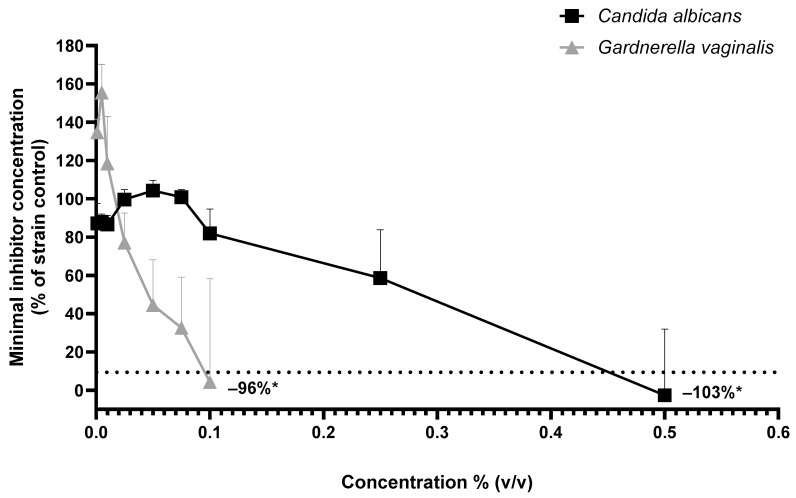
Growth of *Candida albicans* (black square) and *Gardnerella vaginalis* (gray triangle) without cranberry oil and in the presence of a range of concentration of oil from 0.001% to 0.5%, to determine minimum inhibitory concentration. Growth is expressed in percentage of the OD 600 nm of the control culture. Mann–Whitney U-test with * *p* < 0.05.

Minimum inhibitory concentration (MIC) of cranberry oil was evaluated by measuring the OD 600 nm of *C. albicans* and *G. vaginalis*, without oil and with increasing concentration of oil from 0.001% to 0.5% (*v*/*v*) (Figure 6). The MIC of cranberry oil was determined at 0.5% for *C. albicans* with a growth reduction by 103% compared to untreated culture. The MIC of cranberry oil was determined at 0.1% for *G. vaginalis* with a growth reduction by 96% compared to untreated culture.

**Figure 7:**
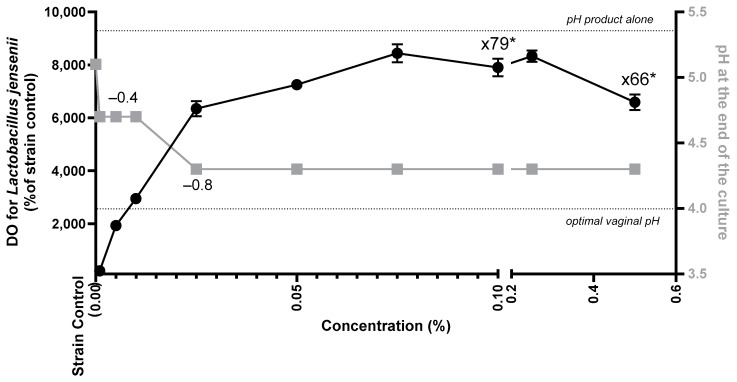
Growth of *Lactobacillus jensenii* based on the OD 600 nm of the culture expressed as a percentage of the control culture without cranberry oil and in the presence of a range of concentrations of the oil, from 0.001% and 0.5% (black dots), and pH of the culture (gray squares). Mann–Whitney U-test with * *p* < 0.05.

Growth of the commensal bacteria *L. jensenii*, a major bacterium of the vulvovaginal microbiota, was observed in the presence of cranberry oil at 0.001% to 0.5% and compared to untreated culture. Cranberry oil significantly increased the growth of *L. jensenii* from 0.001% and reached a maximum between 0.075 and 0.5% of oil (Figure 7). Growth increased by 66-fold to 79-fold. Considering the pH, the pH of cranberry oil was 5.3 and the culture of *L. jensenii* without the oil was 5.1. Immediately after adding cranberry oil, the pH of the culture decreased to 4.7 and the growth of the *L. jensenii* acidified the medium, reaching 4.3, due to the release of organic acids into the culture medium.

### 2.6. Cranberry Oil Favors L. jensenii Growth in a Co-Culture with the Pathogenic C. albicans In Vitro

**Figure 8:**
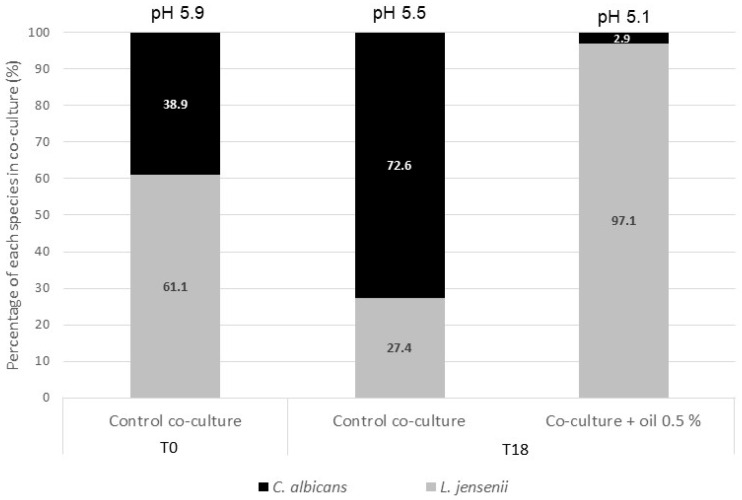
Percentage of *Lactobacillus jensenii* (gray) and *Candida albicans* (black) in a co-culture with or without cranberry oil at 0.5% and pH measurements.

In the previous experiments, cranberry oil was observed to inhibit *C. albicans* and promote *L. jensenii*. A co-culture of both microorganisms was established as a simplified model of vulvovaginal microbiota and cranberry oil was applied in the culture medium at 0.5% (Figure 8). The pH was controlled at 5.9 at the beginning of the culture to favor a dysbiosis condition, i.e., the predominance of *C. albicans*. Proportions of each microbial species were determined based on enumeration values (CFU/mL) at inoculation and after 18 h of incubation. After 18 h without cranberry oil, the ratio was in favor of the pathogenic fungus with 72.6% of *C. albicans* and 27.4% of *L. jensenii* and a culture medium pH at 5.5. Addition of cranberry oil at 0.5% reversed this ratio, so the pathogenic fungus represented only 2.9% of the culture and the pH decreased to 5.1.

### 2.7. Synergistic Anti-Inflammatory Activity of Cranberry Oil and Cranberry Extract on Keratinocytes

**Figure 9:**
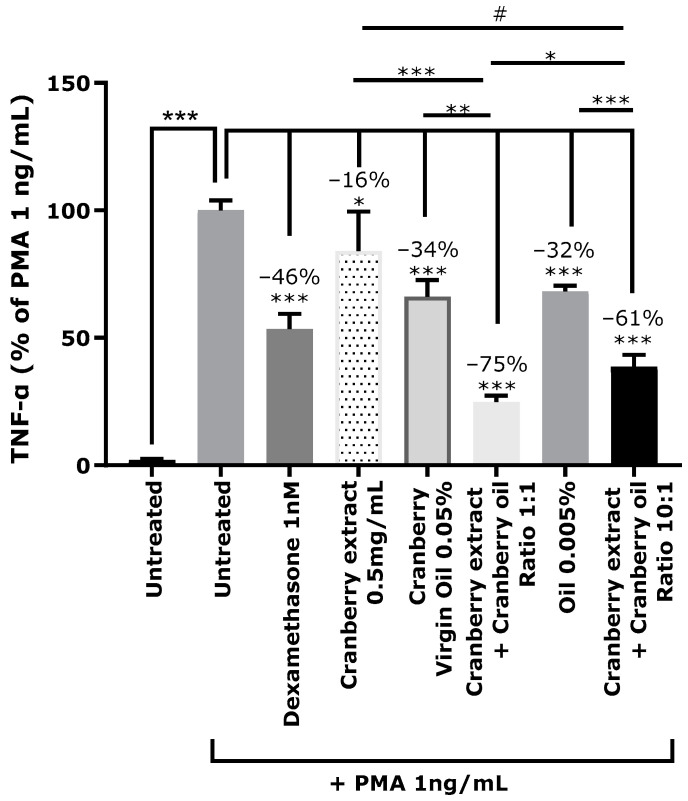
Release of TNF-α (Tumor Necrosis Factor alpha) in normal human epidermal keratinocytes stressed with PMA (Phorbol 12-Myristate 13-Acetate) and pre-treated with anti-inflammatory reference compound dexamethasone at 1 µM (positive control), cranberry extract at 0.5 mg/mL, cranberry oil at 0.05% or 0.05%, or with a combination of cranberry extract 0.5 mg/mL and cranberry oil at 0.05% or 0.005%. Quantification of cytokines is expressed as a percentage of the response of PMA-treated condition. Mann–Whitney U-test, # *p* < 0.1, * *p* < 0.05, ** *p* < 0.01, *** *p* < 0.001.

Inflammation was triggered in NHEKs by PMA at 1 ng/mL, as demonstrated by the release of TNF-α compared to untreated cells (Figure 9). Pre-treatment with dexamethasone, a synthetic anti-inflammatory corticosteroid used as positive control, decreased the release of cytokine in the culture medium by 46%. Cranberry extract at 0.5 mg/mL and cranberry oil 0.05% significantly decreased TNF-α by 16% and 34%, respectively. Combining both cranberry fractions led to a decrease in cytokine release of 75%. Treatment with cranberry extract at 0.5 mg/mL and cranberry oil at 0.005% decreased TNF-α by 61%, while each fraction, applied separately, decreased the cytokine by 16% and 32%, respectively.

## 3. Discussion

*Vaccinium macrocarpon* is a valuable source of bioactive compounds recognized for their health-promoting benefits. The extensive literature describes the role of cranberry flavonoids in the management of urinary tract infections, cardiovascular diseases, and cancers [1,2,3,4]. Cranberry oil emerged as a new ingredient to add value to seeds, a byproduct of agro-industrial processing of berries. The oil has a specific phytochemical profile, primarily because it mainly contains fatty acids, but also some specific phytosterols, differing from the fruit extracts rich in antioxidant and anti-inflammatory flavonoids. Up to now, little research has focused on the biological activity of cranberry oil. Nevertheless, polyunsaturated fatty acids and phytosterols have been demonstrated to promote beneficial flora in vaginal dysbiosis, reduce inflammation, and have antimicrobial properties [16,28,30,35].

This study aimed to evaluate the benefits of cranberry oil in the context of vulvovaginal infection, looking at its antioxidant, anti-inflammatory, and soothing properties, as well as its impact on microbial species associated with vaginal flora. Antioxidant properties were evaluated on NHEKs treated with TBP: oil at 0.05% decreased ROS production by 43%. Shivananda Nayak and collaborators suggested that cranberry oil’s wound-healing properties would be related to polyphenols, or omega-6 and omega-9 fatty acids [24]. Polyunsaturated fatty acids were previously demonstrated to scavenge radicals in human aortic endothelial cells [36]. If total phenols were quantified in cranberry oil, no description of apolar phenolic compounds composing the oil is available in the literature, while other antioxidant compounds have been clearly identified and quantified. Cranberry oil is particularly rich in tocotrienols and, to a lesser extent, in tocopherols, belonging to the vitamin E family. Tocotrienols have attracted less attention than tocopherols, although these molecules display similar antioxidant properties as well as anticancer, antidiabetic, and cardiovascular protective effects [37,38].

We also demonstrated that cranberry oil has anti-inflammatory properties, decreasing the release of TNF-α (−32%) and IL-8 (−33%) in PMA-treated NHEKs. TNF-α, which can be induced by UVB and a range of other inflammatory stimuli, is capable of inducing IL-8 secretion in skin cells [39]. Oxidative stress activates inflammatory pathways through the activation of NF-κB (Nuclear Factor κB) subsequently triggering the production of inflammatory cytokines such as TNF-α, IL-8, and IL-6 [40]. Thus, the antioxidants in cranberry oil may play a role in its anti-inflammatory properties. γ-Tocotrienol, a major tocotrienol in oil, and β-Sitosterol, one of the major phytosterol in cranberry oil, are described as limiting the inflammatory response in keratinocytes by decreasing TNF-α, IL-6, and IL-8 [41,42]. Triterpenoids such as ursolic acid and oleanolic acid are also potent inhibitors of inflammation, targeting NF-κB and TNF-α [43,44]. Further studies will help in deciphering the anti-inflammatory pathway activated by cranberry oil.

In this study, cranberry oil was also demonstrated to lower irritation (−24%) in skin explants exposed to SDS surfactant. Irritation and itching are frequent symptoms induced by variable factors: chemicals such as detergents or conditions such as pathologies as atopic dermatitis, vaginal dysbiosis, and urinary tract infections. Inflammation can play a role in the generation of itching [45]. The epidermis interacts with nerve endings, and the bi-directional cross-talk between the skin and the brain is based on neuropeptides. Under certain conditions, the release of neuropeptides by nerve fibers stimulates keratinocytes to produce interleukins and leads to neurogenic inflammation and itching, impacting quality of life and well-being. Furthermore, neurogenic inflammation has been linked to acute and chronic pathological conditions in the urinary tract [46].

Cranberry oil was demonstrated above to reduce oxidative stress, inflammation, and irritation on keratinocytes and skin explants. These properties are of great interest in sensitive and atopic skin. Inflammation and irritation are symptoms induced by vulvovaginal infections (candidiasis, bacterial vaginosis). In the context of antimicrobial resistance, natural compounds like cranberry oil display interesting properties as potential intimate care ingredients. Cranberry oil was tested on vaginal epithelium stressed with a microbial associated molecular pattern, flagellin, to mimic vaginal dysbiosis. Cranberry oil decreased IL-6 by 36%, confirming anti-inflammatory properties in this context as well.

We went further to explore the potential role of cranberry oil in preventing vulvovaginal dysbiosis by determining MIC on two pathogenic vaginal microorganisms, *C. albicans* and *G. vaginalis*. The effect of cranberry oil on *L. jensenii* growth was also observed. Vaginal health depends, among other factors, on the balance of its microbial flora. The predominance of *Lactobacilli* in vaginal microbiota is considered a health-promoting factor while not being a sine qua non [14]. *Lactobacilli* adhere to vaginal epithelial cells and produce bactericidal bacteriocins and lactic acid, which decrease vaginal pH to 3.5, thus preventing colonization by potential pathogens. *C. albicans* is part of healthy women’s microbiota but is still considered an opportunistic pathogen due to the high prevalence of the fungus in patients suffering from vulvovaginal candidiasis. Bacterial vaginosis is a dysbiosis caused by a reduction in *Lactobacillus* spp. and overgrowth of anaerobic bacteria such as *G. vaginalis*, with the latter being a key component of anaerobic diversity responsible for symptoms of bacterial vaginosis [14,47].

Cranberry oil inhibited growth of *C. albicans* and *G. vaginalis* from 0.25% and 0.025%, respectively. The MIC of cranberry oil was measured at 0.5% (*v*/*v*) for *C. albicans* and 0.1% (*v*/*v*) for *G. vaginalis,* with growth inhibition close to 100%. Standardized cranberry fruit extract has been shown to decrease *C. albicans* biofilms at 0.01% (*w*/*v*), especially the phenolic compound 4-hydroxybenzoic acid [13]. Fractions of cranberry fruit extract have also been demonstrated to decrease the metabolic activity of *C. albicans* from 1% (*w*/*v*). The antimicrobial activity of cranberry seed oil seems to be as efficient as that of the berry extract.

Cranberry oil strongly promoted the growth of the commensal *L. jensenii* from 0.005% (*v*/*v*) and reached an increase of 79-fold at 0.1% (*v*/*v*). Inhibition of *G. vaginalis* and promotion of *L. jensenii* are consistent with the recent findings of Zhu and collaborators [16]. The authors demonstrated, in vitro, that omega-9 oleic acid (18–30% in cranberry oil) selectively promotes the growth of *Lactobacillus* sp. associated with healthy vaginal microbiota while inhibiting bacterial-vaginosis-associated species, such as *Gardnerella* sp. More precisely, oleic acid balanced the population of Lactobacilli in favor of species such as *L. crispatus* and *L. jensenii*, instead of *L. iners*, which is associated with recurring bacterial vaginosis, thanks to oleic-acid-regulated genes *ohya* and *farE*, which are absent in *L. iners*. We conducted a co-culture of *L. jensenii* and *C. albicans* as a simplified model of vaginal microbiota. The pH was intentionally set to 5.9 to mimic dysbiosis, where *C. albicans* is predominant. Cranberry oil favored a high proportion of the lactic acid bacteria (97.1% vs. 27.4% without cranberry oil after 18 h of culture) and induced a pH decrease to 5.1. In vitro assays indicate that cranberry oil is a potent regulator of vaginal flora, mainly due to the promotion of beneficial *L. jensenii* and its antimicrobial activity against *C. albicans* and *G. vaginalis*. As the knowledge on vaginal microbiota progresses with genomic technologies, newly recognized agents associated with vulvovaginal infections or vaginal health are being identified [14]. Further experiments on vaginal *Lactobacilli* and potential pathogenic bacteria and fungi, as well as in vivo studies, are required to confirm these first results.

Cranberry fruit extracts are known for their high antioxidant and anti-inflammatory properties [48]. These extracts are generally representative of the pericarp composition and contain anthocyanins, anthocyanidins, proanthocyanidins, and triterpenoids, mainly ursolic acid [4]. The phytochemical profile of cranberry oil is different, being rich in omega fatty acids, tocochromanols, phytosterols, and triterpenoids, mainly squalene [4,21,25]. A combination of the two types of cranberry extracts would mix a larger set of active molecules, and to our knowledge, this has never been evaluated before. In this study, cranberry oil associated with a fruit extract was tested on a cellular model, and it was observed to act synergistically on inflammation, decreasing TNF-α release by 75% in keratinocytes, while both compounds separately lessened TNF-α release by 34% and 16%, respectively. The synergistic activity of the two cranberry fractions suggests that they could act on different pathways of inflammation. Polyphenols from fruit extract are direct scavengers of ROS, inhibitors of the transcription factor NF-κB and Janus Kinase/signal transducer and activator of transcription (JAK/STAT), and they prevent mitochondrial damage [48]. Further studies are needed to describe the anti-inflammatory pathways targeted by cranberry oil and fruit extract and to explain their synergistic mode of action.

This work demonstrates the potential of cranberry oil in skincare thanks to antioxidant, anti-inflammatory, and soothing properties. Cranberry oil was also evaluated as a potent intimate care ingredient. Anti-inflammatory properties were confirmed on vaginal epithelium and preliminary in vitro evidence indicates that oil favored *Lactobacillus* growth at the expense of pathogenic microorganisms *C. albicans* and *G. vaginalis*, potentially preventing dysbiosis. The initial insight into the synergistic activities of cranberry seed oil and cranberry fruit extract will encourage further studies using ex vivo and in vivo models.

## 4. Materials and Methods

### 4.1. Cranberry Oil and Cranberry Extract

The cranberry oil was obtained via a proprietary solvent-free cold press process of *Vaccinium macrocarpon* seeds upcycled from the juice production. The oil’s fatty acid composition was standardized as follows: linolenic acid (C18:3n3, ω-3) 26–34%, linoleic acid (C18:2n6, ω-6) 32–42%, oleic acid (C18:1n9, ω-9) 18–30%, stearic acid (C18:0) < 2%, palmitic acid (C16:0) 4–7%, eicosenoic acid (C20:1) < 2%, and palmitoleic acid (C16:1) < 1%. Determination of fatty acid distribution was performed by gas chromatography with flame ionization detection (GC-FID) on GC 3800 apparatus (Varian, Tokyo, Japan). The column used was DB-23 60 × 0.25 mm (Agilent, Santa Clara, CA, USA). Oil (100 mg) was diluted in 10 mL MTBE (methyl tert-butyl ether, Sigma, Tokyo, Japan), and 200 µL of this preparation was supplemented with 100 µL of trimethylsulfonium hydroxide solution (Sigma). One microliter was injected for analysis in GC-FID. The cranberry extract was obtained by ethanolic extraction of berries, followed by concentration and spray drying.

### 4.2. Study of Cranberry Oil Antioxidant Activity on Keratinocytes

Antioxidant activity was evaluated by quantification of reactive oxygen species (ROS) in a model of NHEK, which are primary cells freshly isolated from biopsies, pre-treated with an antioxidant, and stressed by TBP solution (Fisher, Waltham, MA, USA). NHEKs were seeded in a black plate with a glass bottom at 20,000 cells per well in 96-well plates with a type I collagen pre-coating in quadruplicate. The cells were incubated for 24 h in complete medium (Epilife medium supplemented with HKGS, Fisher) at 37 °C with 5% CO_2_. After 24 h of culture, the cells were treated in complete medium with the following conditions: cranberry oil 0.05% (*v*/*v*) or resveratrol 200 µM (Sigma), and an antioxidant reference compound used as positive control. Cranberry oil was first diluted to 1% (*v*/*v*) in sterile water supplemented with 1% Tween^®^20 (Sigma), before dilution into complete medium. Skin cells that were untreated and cultivated with complete medium were used as negative control. The cells were then incubated for 24 h at 37 °C, 5% CO_2_.

After 24 h of incubation, detection of ROS was performed. The 2′,7′-Dichlorofluorescin diacetate (DCFH-DA, Sigma) probe was added to the wells at 50 μM for at least 30–40 min at 37 °C. The cells were then washed two times with PBS buffer (Gibco) and treated with the oxidative stress inducer: TBP at 5 mM in PBS buffer. The untreated cells remained in PBS buffer. Finally, the emitted fluorescence was measured in darkness by excitation wavelength at 488 nm and emission wavelength 525 nm using a microplate reader (TECAN).

### 4.3. Study of Cranberry Anti-Inflammatory Activities

#### 4.3.1. Evaluation of Cranberry Oil Anti-Inflammatory Property

Anti-inflammatory activity was evaluated on NHEKs, primary cells isolated from fresh biopsies, by measuring interleukin 8 (IL-8) and Tumor Necrosis Factor alpha (TNF-α) after stress induced by PMA (Sigma). NHEKs were seeded in a type I collagen pre-coated 24-well plate at 30,000 cells per well in triplicate. The cells were incubated for 24 h in complete medium (Epilife medium supplemented with HKGS, Gibco) and 1% of antibiotics (Sigma-Aldrich) at 37 °C with 5% CO_2_. At the end of the incubation, the cells were rinsed twice with PBS and pre-treated with dexamethasone 1 µM (Sigma), a reference anti-inflammatory corticosteroid used as a positive control, or with cranberry oil 0.005% (*v*/*v*) for 24 h, in complete medium with 1% of antibiotics and without hydrocortisone, at 37 °C with 5% CO_2_. Cranberry oil was first diluted at 1% (*v*/*v*) in sterile water supplemented with 1% Tween^®^20, before dilution into the medium.

Untreated skin cells cultivated with complete medium were used as negative control. After 24 h of pre-treatment, the cells were stressed with PMA at 1 ng/mL and incubated for 24 h at 37 °C with 5% CO_2_. At the end of the culture, the cell media were collected and centrifuged at 2,000× *g* for 10 min at 4 °C to eliminate dead cells. The media were stored at −20 °C.

Cellular toxicity of treatments was evaluated through MTT (3-(4,5-dimethylthiazol-2-yl)-2,5-diphenyl-2H-tetrazolium bromide) and also allowed the normalization of IL-8 and TNF-α quantification. MTT solution (Sigma) diluted to 1 mg/mL in basal medium was added to each well and incubated for 3 h at 37 °C and 5% CO_2_. Then, the medium was removed and 300 μL of dimethyl sulfoxide (DMSO, Sigma) was added into each well to dissolve the formazan crystals. Homogenization was performed under orbital agitation for a few minutes. The optical density was measured at a wavelength of 560 nm.

The IL-8 and TNF-α quantification was performed using the Human IL-8 Quantikine ELISA kit (D8000C, R&D Systems, Minneapolis, MI, USA) and Human TNF-alpha Quantikine ELISA kit (DTA00D, R&D Systems), respectively. Briefly, the samples and the standard range were incubated for 2 h in a 96-well plate pre-coated with a monoclonal antibody specific for human IL-8 or TNF-α. After 4 washes with wash buffer provided by the supplier, a polyclonal antibody specific to human IL-8 or TNF-α and conjugated with horseradish peroxidase enzyme (HRP) was incubated for 1 h under orbital agitation. Following 4 washes, a substrate solution was added to the wells for 30 min in the dark. The catalysis of this substrate by HRP generates a blue color in proportion to the amount of IL-8 or TNF-α bound in the initial step. The color development was stopped by a Stop solution, changing it into yellow. The optical density was measured at 450 nm and 540 nm with a microplate reader (TECAN SPARK^®^ 10M). Finally, the four parameter logistic curve analysis was performed via the Myassays website (http://myassays.com) after subtraction of optical density at 450 nm by optical density at 540 nm. The quantification was normalized in relation to the optical density measured via the MTT assay.

#### 4.3.2. Study of Anti-Inflammatory Activity of Cranberry Oil Associated with Cranberry Extract

The anti-inflammatory activity of cranberry oil and cranberry extract was evaluated on NHEKs, primary cells isolated from fresh biopsies, by measurement of TNF-α after stress induced by PMA (Sigma). NHEKs were seeded in a type I collagen pre-coated 24-well plate at 30,000 cells per well in triplicate. The cells were incubated for 24 h in complete medium (Epilife medium supplemented with HKGS, Gibco) and 1% of antibiotics (Sigma-Aldrich) at 37 °C with 5% CO_2_. At the end of the incubation, the cells were rinsed twice with PBS (Gibco) and pre-treated with dexamethasone 1 µM, a reference anti-inflammatory corticosteroid used as a positive control, or with cranberry oil 0.05 or 0.005% (*v*/*v*), or with cranberry extract at 0.5 mg/mL, or with a combination of cranberry extract 0.5 mg/mL with cranberry oil at 0.05 or 0.005% (*v*/*v*). Pre-treatment was conducted for 24 h, in complete medium with 1% of antibiotics and without hydrocortisone, at 37 °C with 5% CO_2_. Cranberry oil was first diluted to 1% (*v*/*v*) in sterile water supplemented with 1% Tween^®^20, before dilution into the medium.

Untreated skin cells, cultivated with complete medium, were used as a negative control. After 24 h of pre-treatment, the cells were stressed with PMA at 1 ng/mL and incubated for 24 h at 37 °C with 5% CO_2_. At the end of the culture, the cell media were collected and centrifuged at 2,000× *g* for 10 min at 4 °C to eliminate dead cells. The media were stored at −20 °C.

Cellular toxicity of treatments was evaluated through MTT (3-(4,5-dimethylthiazol-2-yl)-2,5-diphenyl-2H-tetrazolium bromide) and we also allowed the normalization of TNF-α quantification. MTT solution (Sigma) diluted to 1 mg/mL in basal medium was added to each well and incubated for 3 h at 37 °C, 5% CO_2_. Then, the medium was removed and 300 μL of DMSO (Sigma) was added into each well to dissolve the formazan crystals. Homogenization was performed under orbital agitation for a few minutes. The optical density was measured at a wavelength of 560 nm.

The TNF-α quantification was conducted with the Human TNF-α Quantikine ELISA kit (DTA00D, R&D Systems). Briefly, the samples and the standard range were incubated for 2 h in 96-well plates pre-coated with a monoclonal antibody specific to human TNF-α in the presence of the Assay Diluent RD1F. After 4 washes with wash buffer provided by the supplier, a polyclonal antibody specific for human TNF-α and conjugated with HRP was incubated for 2 h under orbital agitation. Following 4 washes, a substrate solution was added to the wells for 30 min in the dark. The catalysis of this substrate by HRP generates blue color in proportion to the amount of TNF-α bound in the initial step. The color development was stopped by a Stop solution, changing it to yellow color. The optical density was measured at 450 nm and 540 nm with a microplate reader (TECAN SPARK^®^ 10M). Finally, the four parameter logistic curve analysis was performed via the Myassays website (http://myassays.com) after subtracting of optical density at 450 nm from the optical density at 540 nm. The quantification was normalized in relation to the optical density measured via the MTT assay.

### 4.4. Ex Vivo Evaluation of Cranberry Oil Anti-Inflammatory Activity on Reconstructed Human Vaginal Epithelium

Anti-inflammatory activity was evaluated on EpiVaginal™ tissue model (Mattek) stressed with flagellin, a bacterial protein involved in cell mobility. EpiVaginal™ tissues are based on human-derived Vaginal EctoCervical (VEC) epithelial and dendritic cells. Upon receipt, EpiVaginal™ tissues were transferred into 6-well plates with 1 mL of dedicated Maintenance Medium (Mattek) for pre-equilibration. After 6 h, EpiVaginal™ tissues were incubated with 900 μL of VEC media without hydrocortisone (Mattek) and topically pre-treated with 100 μL of cranberry oil 0.05%. Cranberry oil was first diluted to 1% (*v*/*v*) in sterile water supplemented with 1% Tween^®^20 and then diluted into the medium. EpiVaginal™ was incubated for 24 h at 37 °C with 5% CO_2_. At the end of the incubation, the media were removed and the wells were rinsed twice with PBS (Gibco). Then, EpiVaginal™ tissues were stressed with flagellin at 100 ng/mL (Sigma) in the hydrocortisone-free media and incubated for 24 h at 37 °C with 5% CO_2_. Untreated tissues were used as a negative control. At the end of the culture, the cell media were collected and centrifuged at 2000× *g* for 10 min at 4 °C to remove dead cells. The media were stored at −20 °C before quantification of the IL-6.

The IL-6 quantification was realized with the Human Luminex Human Discovery Assay ELISA kit (LXSAHM-04, R&D Systems). Briefly, the samples and the standard range were incubated for 2 h in the dark in 96-well dark plates in the presence of microparticle cocktail magnetic beads. During the fourth wash with the wash buffer provided by the supplier, beads were held by a magnetic plate. Then, a Biotin-antibody specific for human IL-6 was incubated for 1 h under orbital agitation. Following 4 washes on the magnetic plate, a Streptavidin solution was added to the wells for 30 min. Following 4 washes on the magnetic plate, the beads were suspended in wash buffer before quantification of IL-6 (MagPix, Luminex^®^).

### 4.5. Anti-Irritation Activity of Cranberry Oil on Skin Explants

Skin explants treated with SDS were used to mimic irritation conditions. For this model, skin explants from two independent donors, aged 45 and 19 years old, were utilized. Upon receipt, the skin was cleaned and disinfected through successive baths in ethanol and Epilife medium supplemented with HKGS factor and antibiotics, including antifungal agents. For each donor, skin samples were cut using a 6 mm circular biopsy punch. The skin explants were placed in 24-well plates on a 3D-bioprinted specific support (3D Morphoz). An air–liquid interface was created by adding skin culture medium in the bottom of the wells (Givaudan, Vernier, Switzerland). Four explants per condition were cultured and incubated overnight at 37 °C with 5% CO_2_. The next morning, irritation was induced by a 10 μL topical application of SDS at 0.2% (*w*/*v*) for 40 min at 37 °C with 5% CO_2_. After 40 min, the skin explants were rinsed twice with PBS (phosphate buffer saline) and treated or not with cranberry oil 0.5% (*v*/*v*) before being incubated again with renewed skin culture medium. Cranberry oil was diluted in sterile water with 1% Tween^®^20. The skin explants treated with PBS served as untreated and non-irritated controls.

Induction of irritation was applied twice a day (in the morning and at the end of the afternoon) for 4 days to mimic the irritation induced by the daily use of detergent bases. After the last induction of irritation, the skin explants were rinsed twice with PBS before being fixed in formalin 10%.

After fixation, skin explants were dehydrated through successive baths of increasing ethanol concentration, followed by xylene and, finally, paraffin. This step allows the replacement of the water in the skin explants with a paraffin filling. The skin explants were then embedded in paraffin blocs and cut into 4 µm thick slices using a microtome. After drying, the slices were dewaxed and stained with a hematoxylin and eosin kit (Leica) to observe the general morphology of the skin after the various treatments.

Pictures were taken for each skin slice with an inverted Zeiss microscope coupled to Zen Blue software version 2.5. For each image, the irritation was scored by five experts following this scale: (1) no irritation, (2) low irritation associated with slight edema, (3) medium irritation associated with edema in only one skin layer, and (4) high irritation associated with edema in both the dermis and epidermis.

### 4.6. Effect of Cranberry Oil on Growth of Pathogenic and Commensal Microorganisms of the Vulvovaginal Flora In Vitro

To study the impact of cranberry oil on the vulvovaginal flora, we chose a microbial species involved in bacterial vaginitis, *Gardnerella vaginalis* (DSM 4944 isolated from vaginal secretions), and a fungus implicated in candidiasis, *Candida albicans* (DSM 3454 isolated from the vagina) [14,47]. A lactic acid bacterium, *Lactobacillus jensenii* (DSM 20557 isolated from vaginal discharge), was selected as a representative of *Lactobacilli*, which predominates in healthy vaginal microbiota. *G. vaginalis* and *C. albicans* were grown in Mueller–Hinton (MH) medium, and *L. jensenii* was grown in thioglycolate at pH 6.02, as the strain’s growth is inhibited at higher pH values. The strains were subcultured twice at 37 °C before testing. MH medium was selected for the experiments due to its neutral pH of 7.2, which is drastically different from the healthy vaginal pH of 3.8–4.5. A neutral pH mimics the conditions of dysbiosis in the vulvovaginal microbiota.

Cranberry oil was tested in the range of 0.005% to 0.5% (*v*/*v*), and its vehicle, sterile water supplemented with 1% Tween^®^20, was tested in the range of 0.005% to 0.25% (*v*/*v*). Both products were solubilized in pure water to prepare a stock solution, and then the dilutions were carried out in pure water for *C. albicans* and *G. vaginalis*, or in thioglycolate for *L. jensenii*.

The minimum inhibitory concentration (MIC) of cranberry oil was determined for the three strains. The ISO 20776-1: 2019 (Reference method of microdilution in broth) [49] was followed to determine the MIC of cranberry oil for each strain. Cranberry oil dilutions were added to microplate wells in triplicate for each concentration (100 µL per well). Bacterial suspensions were adjusted to between 1 and 8 × 10^5^ CFU/mL, and 100 μL were added per well. The bacterial suspension was also cultured with pure water or thioglycolate, as a positive control for strain growth. Negative controls were performed with cranberry oil alone and medium alone. The plates were incubated under anaerobic conditions for *G. vaginalis* for 48 h ± 2 h. For *C. albicans*, the plates were incubated in aerobic conditions for 18 h ± 2 h. For *L. jensenii*, the plates were incubated in anaerobic conditions for 42 h. After incubation, growth was evaluated by measuring absorbance at 600 nm for each well after shaking the plates. In parallel, the pH was measured for the culture of *L. jensenii* using a pH paper strip.

### 4.7. Co-Culture of Lactobacillus jensenii and Candida albicans to Model Vulvovaginal Microbiota; Effect of Cranberry Oil on Bacterial Growth

*C. albicans* and *L. jensenii* were subcultured twice from stock solutions to thioglycolate broth and incubated at 37 °C before the tests. Cranberry oil was prepared in thioglycolate broth to reach a final concentration of 0.5% (*v*/*v*) per well. Enumeration was performed at T0 and T18 h, in wells inoculated with 100 µL of microbial suspension and 100 µL of thioglycolate medium at pH 6. Negative controls were conducted with 100 µL of cranberry oil 0.5% (*v*/*v*) and 100 µL of thioglycolate medium at pH 6, and 200 µL of thioglycolate medium at pH 6. Each condition was repeated three times on the microplate (3 wells).

Initial cell suspensions of *C. albicans* and *L. jensenii* were prepared in thioglycolate broth at pH 6, and the cell concentration was adjusted to 10^5 CFU/mL. A standardized culture of each strain was placed in the wells of a 96-well microplate in triplicate at a ratio of 1:1. Cranberry oil at 0.5% was added to the wells (100 µL). The plates were incubated under anaerobic conditions at 37 °C for 18 h.

*C. albicans* colonies and *L. jensenii* were counted on BHI agar after 24 h and 48 h of incubation, respectively, at 37 °C under anaerobic conditions. The pH was monitored in parallel using a pH paper strip (Macherey Nagel, Range 3.1–8.3).

### 4.8. Statistical Analysis

For all studies, a Shapiro–Wilk test was performed to verify whether the raw data followed the Gaussian distribution. In the case of normally distributed data, the mean values were compared using an unpaired Student’s *t*-test. For non-normally distributed data, a Mann–Whitney U-test was conducted. Regardless of the statistical test used, we considered significant results as follows: # *p* < 0.1, * *p* < 0.05, ** *p* < 0.01, and *** *p* < 0.001.

## Data Availability

The original contributions presented in the study are included in the article; further inquiries can be directed to the corresponding author.
